# Haemochromatosis in a kidney transplant recipient: a case report

**DOI:** 10.1186/s12882-021-02416-9

**Published:** 2021-05-29

**Authors:** Izabela Zakrocka, Iwona Baranowicz-Gąszczyk, Wojciech Załuska

**Affiliations:** Department of Nephrology, Medical University of Lublin, Jaczewskiego street 8, 20-090 Lublin, Poland

**Keywords:** Haemochromatosis, Kidney transplant, Case report, Iron, Tacrolimus

## Abstract

**Background:**

Iron overload is inevitably related to chronic kidney disease (CKD) treatment. Haemochromatosis leads to multiorgan damage and is associated with increased mortality. Primary haemochromatosis is the most common autosomal recessive disease in white populations. In most cases, the classic form of hereditary haemochromatosis is caused by mutations, mainly C282Y and H63D, in the haemochromatosis gene (HFE). Secondary haemochromatosis can be triggered by iron administration and blood transfusions. Haemochromatosis is rarely reported in kidney transplant recipients. Atypical factors may evoke haemochromatosis in patients without HFE mutations or other standard risk factors.

**Case presentation:**

In the current study, we present a patient who started to have haemochromatosis symptoms after kidney transplantation. A 37-year-old man after kidney transplantation from a deceased donor was admitted to the hospital due to high serum ferritin levels and impaired graft function. The patient’s past medical history included arterial hypertension, embolization of both renal arteries and necrosis of the left femoral head. Glomerulonephritis was suspected as a cause of CKD; however, severe kidney failure was diagnosed, kidney biopsy was not performed, and the patient started intermittent haemodialysis. While on dialysis to treat anaemia, the patient had received erythropoietin and iron intravenously, and the maximal serum ferritin level was 2115 ng/ml. After kidney transplantation, ferritin levels started to increase rapidly, with a maximum level of 9468 ng/ml one and a half years after surgery. His genetic study showed HFE C282Y heterozygosity. Symptoms of haemochromatosis, such as skin hyperpigmentation, elevated activity of aminotransferases, impaired glucose tolerance and heart failure, were observed. Therapeutic phlebotomy was started, and 36 procedures were performed. After treatment, graft function significantly improved, most haemochromatosis symptoms resolved, and the serum ferritin level significantly decreased.

**Conclusions:**

Haemochromatosis can occur in heterozygotic HFE patients after kidney transplantation. Iron administration, infections, type of immunosuppression and liver dysfunction should be considered potential triggers of haemochromatosis in this group of patients.

## Background

Haemochromatosis is an iron storage disorder leading to multiple organ damage and premature death [1]. Heavy iron overload leads to various complications, including liver cirrhosis, arthritis, cardiomyopathy, arrhythmias, pericarditis, diabetes, thyroid gland dysfunction, hypogonadotropic hypogonadism, immunosuppression and skin discolouration [1, 2]. Primary haemochromatosis is the most common autosomal recessive disorder in northern populations, with a prevalence of 1 in 300 individuals [2]. Mutations in HFE, including C282Y and H63D, are responsible for most cases of primary haemochromatosis [2]. Men are affected two to three times more often than women and start to present haemochromatosis symptoms in the fifth decade [3]. Whereas the majority of patients with haemochromatosis are homozygotes for C282Y, less than 5% are C282Y or H63D heterozygotes [2]. Iron disorders caused by other gene mutations, such as haemojuvelin, hepcidin antimicrobial peptide, transferrin receptor-2 and ferroportin, are very rare and occur at a younger age [1]. Although heterozygotic C282Y patients do not generally develop haemochromatosis symptoms, under some conditions, such as liver steatosis, diabetes or excessive alcohol consumption, these symptoms may become more prevalent [4, 5]. Iatrogenic iron overload and blood transfusions are the main causes of secondary haemochromatosis in patients with CKD [6]. Since ferritin is an acute phase protein, its high serum level can be observed not only after excessive iron administration but also in patients with liver injury, systemic inflammatory conditions, haemoglobinopathies and haemodialysis treatment [7, 8]. However, transferrin saturation (TS) above 45% and a serum ferritin concentration above 300 ng/ml or unexplained multiorgan damage with liver involvement indicate the need for haemochromatosis testing [1]. The presence of HFE mutation is related to reduced responsiveness to the treatment [9]. Despite common clinical problem, reports about haemochromatosis in kidney transplant patients are rare [9, 10]. This study presents a case of a kidney transplant recipient with haemochromatosis diagnosed after kidney transplantation and treated through therapeutic phlebotomy.

## Case presentation

**Fig. 1:**
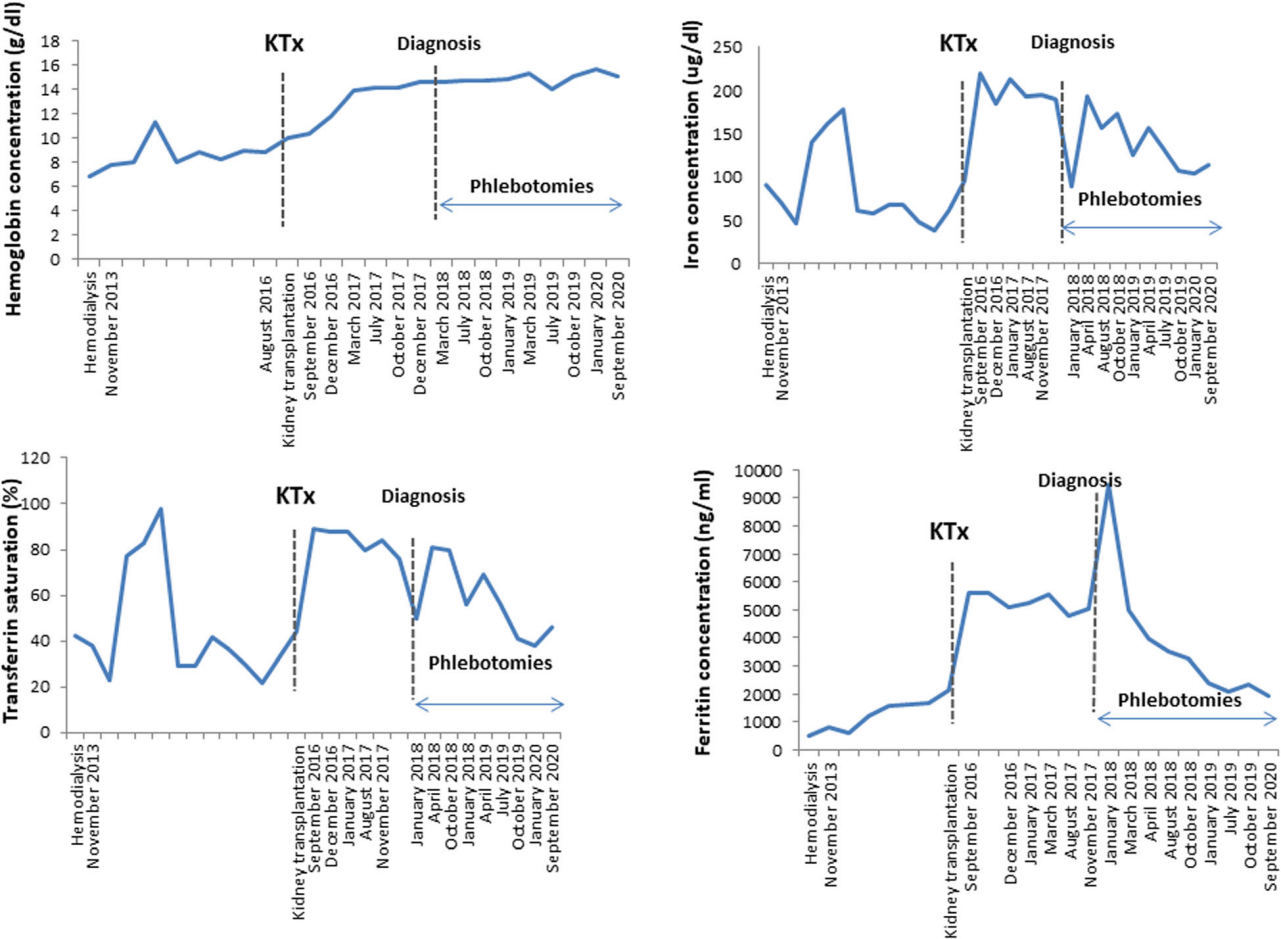
Haemoglobin concentration (g/dl), iron concentration (μg/dl), transferrin saturation (%) and ferritin concentration (ng/ml) in 7 year follow up. Kidney transplantation was performed in August 2016

**Fig. 2:**
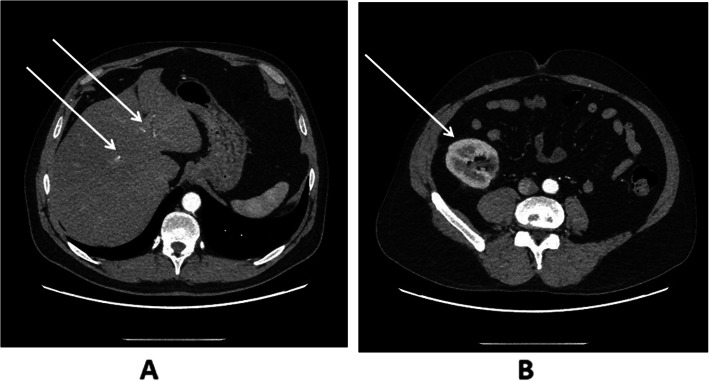
Computed tomography (CT) of the abdomen. Liver steatosis (white arrows) (Fig. 2 A). Transplanted kidney in the right iliac fossa (white arrow), 116 × 62 mm size, without abnormalities (Fig. 2 B)

A 37-year-old recipient of a renal transplant from a deceased donor was admitted to the Nephrology Clinic due to high serum ferritin levels and impaired graft function. CKD was diagnosed 3 years prior to transplantation, and glomerulonephritis was suspected; however, CKD progressed to its end stage, and haemodialysis was started. Arterial hypertension, secondary anaemia and hyperparathyroidism were diagnosed. The patient underwent bilateral renal artery embolization due to resistant hypertension. As a complication of renal angioembolization, patient was diagnosed with left femoral head necrosis, which was successfully treated conservatively, patient did not report any symptoms in later observation. No gastrointestinal tract diseases were diagnosed, alcohol dependence was excluded, and tests for hepatitis B virus, hepatitis C virus, cytomegalovirus (CMV) and Epstein-Barr virus infection were negative. During 33 months of dialysis, the patient received 8200 mg of iron intravenously (from 200 mg to 500 mg per month) and two blood units for the treatment of secondary anaemia. His TS and ferritin level varied, with maximal values reported shortly before kidney transplantation of 44% and 2115 ng/ml, respectively (Fig. 1), but no symptoms of iron overload were found. Kidney transplantation was performed without significant complications, but a slight impairment of graft function was observed (creatinine, 1.6 mg/dl − 1.99 mg/dl). For immunosuppression, the patient received mycophenolate mofetil, tacrolimus, and prednisone. Two months after kidney transplantation, the level of ferritin was 5624 ng/ml, with concomitant anaemia (haemoglobin concentration, 10.4 g/dl), TS 89% and no iron supplements. CMV infection and urinary tract infection caused by *Enterococcus faecalis* were diagnosed and treated appropriately. Eighteen months after kidney transplantation, the haemochromatosis suspicion was raised, since the ferritin concentration rose to 9468 ng/ml and signs of liver damage were observed. The activity level of alanine aminotransferase was 50–134 U/l (normal range < 41 U/l), aspartate aminotransferase was 40–59 U/l (normal range < 34 U/l), gamma-glutamyl transferase was 384 U/l (normal range < 50 U/l), alkaline phosphatase was 134 U/l (limit 129 U/l) and amylase was 85 U/l (limit 53 U/l). Infection markers (including CMV DNA) and cancer markers were negative. Gastroscopy and colonoscopy did not reveal any pathology. The haptoglobin concentration was within the normal range, excluding intravascular haemolysis. Wilson’s disease was ruled out after ceruloplasmin level analysis. The patient reported poor exercise tolerance, darkening of the skin was observed on physical examination, and impaired glucose tolerance was diagnosed. Computed tomography of the abdomen revealed liver steatosis (Fig. 2 A), and the transplanted kidney located in the right iliac fossa had no pathologies (Fig. 2 B). In echocardiography, left ventricular hypertrophy was found, with an ejection fraction of 62%. Due to calcifications observed in the left anterior descending artery, the patient underwent percutaneous coronary intervention. Since a low haemoglobin level persisted (10.9 g/dl – 11.3 g/dl), darbopoetin treatment was started. Because hepatic laboratory test results improved, liver biopsy was not performed. Genetic tests revealed that the patient was heterozygous for C282Y. After haematological consultation, the decision was made to start therapeutic phlebotomy. The patient received necessary information about the proposed methods of treatment and their complications. When informed consent was obtained from the patient, phlebotomy was started, and the patient tolerated the procedure very well. After 36 phlebotomies, the ferritin concentration decreased to 1921 ng/ml, TS decreased to 46%, the haemoglobin level increased to 15.1 g/dl and kidney function significantly improved (creatinine 1.3 mg/dl). At the time of writing this manuscript, graft function remained stable, and most symptoms of haemochromatosis gradually deteriorated after phlebotomy.

## Discussion and conclusions

Iron overload is an important problem in kidney transplant recipients. Supplementation with iron secondary to erythropoiesis-stimulating agent administration in patients with CKD or on haemodialysis may have serious clinical implications. According to Lorenz et al., high serum ferritin levels and TS were observed in 9.4% of patients after kidney transplantation [9]. Among them 36.3% of patients had mutations in HFE. On the other hand, Ramirez et al. reported that the prevalence of HFE mutations, although high in the general population, was not always associated with laboratory and clinical findings leading to a haemochromatosis diagnosis [11]. Additionally, patients with evident criteria of haemochromatosis after kidney transplantation may show neither of the mutations related to the disease [10]. Moreover, some patients may even have high TS or ferritin levels, suggesting iron overload, although they did not receive the maximal iron dose during anaemia treatment [9]. Systemic inflammation, malignancies, liver damage, viral infections (caused by hepatitis B virus, hepatitis C virus, CMV) and alcohol consumption are claimed to be responsible for the iron overload phenotype [9] and in patients with C282Y/H63D heterozygosity [1]. A similar observation was made by Yaprak et al. [12]. In a study by Jorge et al., it was suggested that normalization of kidney endocrine function, especially in terms of increased erythropoietin production, and a lack of uraemic toxins may rapidly evoke haemochromatosis [10]. Other factors, such as surgery itself and immunosuppressive drug administration, may also lead to immune imbalance and iron metabolism abnormalities. In a recent study, Akhtar et al. observed tacrolimus-induced iron overload in the livers of Wistar rats, mostly due to increased expression of hepcidin and immunological system activation [13]. In our patient, one of the risk factors related to haemochromatosis manifestation was liver steatosis, which was diagnosed after kidney transplantation, probably as a side effect of immunosuppression. Second, infections after kidney transplantation, both viral and bacterial, could be potential triggers of haemochromatosis in the present case. Iron administration, although at high doses, did not evoke a haemochromatosis phenotype in our patient when he was haemodialyzed, despite elevated serum ferritin levels and high TS.

Phlebotomy is the method of choice in the treatment of haemochromatosis. Other therapies, namely, erythrocytapheresis and iron binders such as desferroxamine mesylate or deferasirox, are considered alternative treatments [2]. In patients with liver cirrhosis or hepatocellular carcinoma, liver transplantation may be the only method of treatment [14]. Phlebotomy procedures are safe and effective, with tiredness as a main side effect [15]. The goal of the therapy is lowering serum ferritin levels below 50 ng/ml, with haemoglobin levels maintained above 11 g/dl [1]. Most symptoms of haemochromatosis, including chronic fatigue and liver damage, were shown to disappear after phlebotomy; however, arthropathy, pancreatic endocrine dysfunction and hypogonadism have a less robust response to this therapy [1, 2]. Similarly, in our patient, haemochromatosis symptoms diminished after multiple phlebotomies, serum ferritin was significantly lowered, and haemoglobin levels improved.

A limitation of our study is that only HFE mutations were analysed, since testing of other genes was not available in our laboratory. However, because non-HFE mutations occur in different age groups, they were not taken under consideration for our patient.

Our study presents the case of a post-kidney transplantation patient in whom haemochromatosis symptoms became clinically apparent after kidney transplantation. Repeated phlebotomies significantly improved the haemochromatosis course and prevented disease complications. Since reports about haemochromatosis in kidney transplant recipients are rare and their results remain inconclusive, more information is needed about haemochromatosis pathogenesis in this group of patients. Special care is necessary for CKD and kidney transplant patients to prevent iron overload and its consequences.

## Data Availability

The datasets used and analysed during the current study are available from the corresponding author on reasonable request.
